# Increased Dose in Spine Stereotactic Radiosurgery for Metastatic Disease: Are We Underestimating the Risks?

**DOI:** 10.3390/medicina60091514

**Published:** 2024-09-17

**Authors:** Gil Kimchi, Maya Nulman, Saeda Haj, Idan Bar-Orian, Ory Haisraely, Ran Harel

**Affiliations:** 1 Department of Neurological Surgery, Sheba Medical Center, Ramat Gan 5262000, Israel; gilkimchi1@gmail.com (G.K.); mayanulman@gmail.com (M.N.); 2 Faculty of Medical and Health Sciences, Tel-Aviv University, Tel Aviv 6139001, Israel; idan.barorian@sheba.health.gov.il; 3 Department of Orthopedics B and Spine Surgery, Galilee Medical Center, Nahariya 2210001, Israel; 4 Radiation Oncology Unit, Oncology Institute, Sheba Medical Center, Ramat Gan 5262000, Israel; orihaisraely@gmail.com

**Keywords:** spine tumors, spine radiosurgery, stereotactic body radiotherapy, guidelines, radiation dose

## Abstract

*Background and Objectives*: The recently published Spine Stereotactic Radiosurgery (SSRS) ESTRO guidelines advise against treating spinal metastatic disease with a single dose equal to or smaller than 18 Gy, prioritizing local control over the potential for complications. This study aims to assess the necessity and validity of these higher dose recommendations by evaluating the outcomes and experiences with lower radiation doses. *Materials and Methods*: A retrospective evaluation of SSRS patients treated at a single institute was conducted. The outcomes and complications of this cohort were compared to the current literature and the data supporting the new ESTRO guidelines. *Results*: A total of 149 treatment sessions involving 242 spinal levels were evaluated. The overall local control rate was 91.2%. The mean radiation dose for the local control group compared to the local failure group was similar (17.5 vs. 17.6 Gy, not significant). The overall complication rate was 6%. These results are consistent with previous publications evaluating SSRS for metastatic spinal disease. *Conclusions*: SSRS dose escalation may increase local control efficacy but comes with a higher risk of complications. The evidence supporting the strong recommendations in the recent ESTRO guidelines is not robust enough to justify a universal application. Given the palliative nature of treatment for metastatic patients, dose determination should be individualized based on patient conditions and preferences, with a detailed discussion about the risk–benefit ratio of increased doses and the level of evidence supporting these recommendations.

## 1. Introduction

In a recently published reflection on the development of the monumental Timmerman constraints, Dr. Timmerman admits the following: “In all honesty, except for the spinal cord, all other limits for this original table were my educated guesses”. These constraints have been, and continue to be, pivotal for spine stereotactic body radiotherapy (SBRT) planning and guiding clinical trials. However, they were based for a prolonged period on the shared notion that these restrictions were absolute, despite lacking a strong basis to support them [1]. Recently published European Society for Radiotherapy and Oncology (ESTRO) guidelines for spinal stereotactic radiosurgery (SSRS) recommend a treatment dose higher than 18 Gy in one fraction or its equivalent [2]. These new guidelines advise against SSRS doses of 16 or 18 Gy, despite numerous studies demonstrating that these lower doses can be both effective and safe. This discrepancy raises important questions about the basis for these recommendations and whether the potential risks of higher doses have been fully considered. This paper aims to critically evaluate the evidence supporting these new guidelines and assess whether the risk–benefit ratio has been adequately addressed. It will present the senior author’s institutional results of 18 Gy SSRS and compare them with the current literature to evaluate the necessity of complying with the higher dose recommendations.

## 2. Materials and Methods

This is a retrospective study of patient records of those treated with SSRS at Sheba Medical Center, Ramat-Gan, Israel by the senior author. After the study was approved by the Institutional Review Board (approval code: 1555-14-SMC, approved on 26 November 2014), the authors reviewed the records of patients treated between September 2011 and February 2023.

### 2.1. SSRS Technique and Constraints

All patients were immobilized supine in either a head-to-shoulders thermoplastic mask (for tumors involving occiput to T5) or vacuum cushions (for lesions involving T6 to sacrum). All patients were scanned using 1.5 mm thick contiguous CT slices using a large bore CT simulator. Additionally, unless there was a contraindication to MR imaging, all patients underwent spine MRI with axial turbo spin echo T1-weighted images without and with Gadolinium, T2 scans, and axial STIR images to optimize tumor delineation. CT myelograms were performed in patients who could not undergo MRI scans or those with incomplete visualization of the neural structures or thecal sac related to instrument artifact. MRI and CT image datasets were then imported into the iPlannet treatment planning system (TPS) (BrainLab^®^, Feldkirchen, Germany) and rigorously fused. The Clinical Target Volume (CTV) and critical structures (cord or thecal sac in the area of the cauda; lungs, kidneys, bowel, and esophagus) were contoured according to the International Spine Radiosurgery Consortium Consensus Guidelines for Target Volume Definition. Spinal cord contouring was extended 4.5 mm (three CT slices) rostrally and caudally beyond the CTV in all cases. No margins were added (PTV = CTV). In the first 3 cases a plan involving 7 coplanar beams using the step and shoot IMRT technique was generated with brain lab iPlannet planning software (Version 3.0.0). All subsequent cases were planned using the Eclipse VMAT algorithm (Rapid Arc, Eclipse, Varian, CA, USA). The plan was exported from the iPlannet to the Eclipse work station. The plan was calculated using 2–3 arcs of 360 degrees. The image fusion, contouring, and plan were evaluated by both the planning physician, the radiation oncologists, and the radiation physicists. The prescription dose was typically 16 Gy (to cover one patient) in the first 35 sessions. Most sessions were treated with 18 Gy to at least 90% of the CTV. Cord constraint was set at no more than 10% of the contoured volume or absolute 0.35 cc to receive 10 Gy or above. The maximum cord point dose (Dmax) was limited to 14 Gy. The inverse planning algorithms used robust planning software, which accounted for instrument-related scatter and allowed for more accurate tumor and spinal cord dosing. On the day of treatment, the patient was positioned according to simulation markings. Using the ExacTrac (Brainlab, Munich, Germany) on-board imaging system, two diagonal stereo X-ray images were obtained and fused to two digitally reconstructed radiographs (DRRs) generated in the same orientation from the simulation CT. Shifts were made in the x, y, and z directions as needed and another set of X-ray images were obtained to confirm final patient positioning. In select cases, on-board cone beam computerized tomography (CBCT) was used to verify position, particularly in the mid-thoracic region, as the vertebral bodies resemble each other and the ExacTrac fusion algorithm can achieve an erroneous, but seemingly acceptable fusion of proximal levels. Treatment was delivered using the Novalis linear accelerator with a micro-multileaf collimator for beam shaping (Novalis, Brainlab). All patients were monitored during treatment using ExacTrac (Brainlab) [3].

### 2.2. Follow-Up and Outcome Evaluation

For local control evaluation, patients had undergone serial follow-up MRI examination. Most metastatic patients had MRI examination every three months, slow-growing metastases were imaged every four months (sarcoma, chordoma), and patients with nerve sheath tumors or meningiomas had serial MRI every six months. MRIs were evaluated by the senior author and by a multi-disciplinary team during institutional tumor board meetings and classified as local control if the tumor had not increased in size. A physical exam and adverse events were recorded in patients’ charts and reported during tumor board meetings.

Demographics and outcomes were compared between the local failure and local control groups using the Student t-test and Chi-square test. A local failure Kaplan–Meier curve was generated (SPSS 23, IBM, Armonk, NY, USA).

## 3. Results

**Figure 1:**
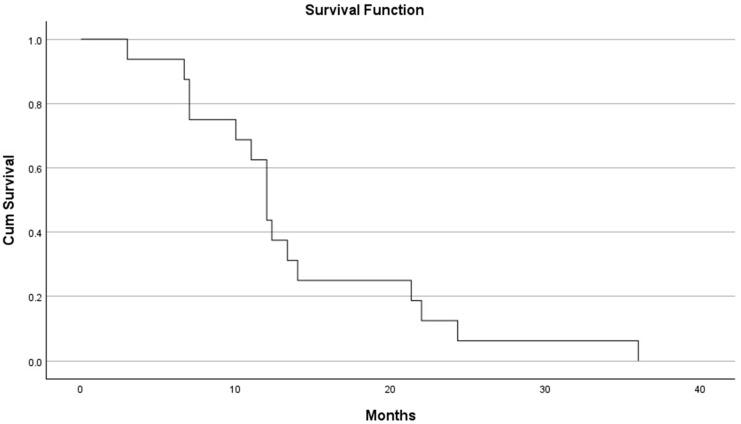
Kaplan–Meier curve for local recurrence duration (months).

Overall, one hundred and sixteen patients were treated in 149 treatment sessions, including 166 lesions in 242 spinal levels. The local control rate was 91.2%. Table 1 depicts the demographic and pathological characteristics of the cohort, stratified to local failure vs. local control groups. The mean dose for both cohorts was 17.6 Gy and 17.5 Gy (local failure and local control groups, respectively (*p* > 0.7)). The only significant difference between the groups was a longer mean follow-up duration for the local failure patients (40 vs. 26 months, *p* = 0.005). Figure 1 depicts the Kaplan–Meier curve for local recurrence time.

### Post-Treatment Complications

The overall complication rate was 6%. Complications included two cases of transient swallowing difficulties (CTCAE Grade 1). Four patients experienced worsening pathological fractures: two were treated with kyphoplasty (CTCAE Grade 2), one with pedicle fixation (CTCAE Grade 3), and one was managed conservatively (CTCAE Grade 1). One patient treated for C5 thyroid carcinoma metastasis with C5 corpectomy and instrumented fusion followed by SRS initially responded well but developed an esophageal perforation 16 months post-SRS, exposing a plate and screw, necessitating surgical intervention (CTCAE Grade 4). Another patient, previously operated on for T11 metastases from renal cell carcinoma via a lateral transthoracic approach, received SRS for metastases at T4 and T6. This patient initially responded well but developed esophageal bleeding requiring surgical intervention seven months post-SRS (CTCAE Grade 4). One patient developed post-operative polyneuropathy following SRS to a metastasis in the lumbar vertebra and psoas muscle (CTCAE Grade 2). None of the patients developed radiation-induced myelopathy, and none required steroid treatment for more than three days [4].

## 4. Discussion

Treatment guidelines are essential for directing clinical practice, providing a framework for consistent and evidence-based patient care. However, it is crucial for individual clinicians to understand the evidence behind these guidelines to make informed and judicious decisions. The new ESTRO guidelines advise against treating spinal metastatic disease with a single dose equal to or smaller than 18 Gy, prioritizing local control over the potential for complications. This study aims to assess the necessity and validity of these higher dose recommendations by evaluating the outcomes and experiences with lower radiation doses. With the spine being one of the most common sites for metastatic spread [5,6], the management of spinal metastases remains a critical concern due to the need to balance effective local control with the risk of complications. In 2011, Hall et al. [7] published a comprehensive review of 1775 treated lesions using SSRS, with doses ranging from 8 to 25 Gy in a single fraction or various hypofractionation schemes. The reported local control rates were around 90%, with a very low risk of myelopathy, demonstrating the efficacy and safety of SSRS. Despite these promising results, the debate over the optimal SSRS dose has persisted. Different institutions have adopted varying dosing strategies, with some favoring relatively lower doses of 14–18 Gy in a single fraction, while others have embraced higher doses such as 24 Gy in a single fraction. For pathologies with higher radioresistance, the need for increased doses to achieve control is well established. Bishop et al. [8] studied metastatic sarcoma patterns of recurrence following SSRS and concluded that higher doses yield better local control. Additionally, a cohort of renal cell carcinoma metastasis patients treated with either a single fraction of 24 Gy or multi-fraction regimens (27 Gy in three fractions or 30 Gy in five fractions) demonstrated improved local control rates in the high-dose single fraction arm. Notably, the 2-year local control rate in this arm was 86% [9].

Yamada et al. reported the results from a single institution, comparing local control rates based on prescribed doses [10]. Initially, in 2003, 62 lesions were treated with a low dose (GTV D95 up to 18.3 Gy). Subsequently, 749 lesions were treated following dose escalation (median dose 23.56 Gy). The low-dose group experienced a 14% recurrence rate, while the high-dose group had a significantly lower recurrence rate of 2.1% at 2 years. However, two patients in the high-dose group developed Grade 3 myelopathy, and other complications were not reported. Despite the significant findings, these results should be interpreted with caution. The low-dose group was treated during the early learning curve phase, with inferior planning technology, prior to the establishment of tumor contouring guidelines, and had a longer follow-up period. Moreover, the local control rates in Yamada’s cohort are superior to those reported by Ghia et al. [9] despite similar doses, suggesting a variation in the planning strategy. This discrepancy suggests that factors other than dose alone, such as technological advancements and improved treatment protocols, may play a crucial role in outcomes. Furthermore, any oncological treatment decision should balance efficacy against the risk of complications, which were not comprehensively reported in Yamada’s study.

A randomized trial evaluating SSRS (16 or 18 Gy in one fraction) compared to conventional radiation (8 Gy in one fraction) revealed no significant difference in pain control or side effects at 3 months (RTOG 0631) [11]. Unfortunately, this study did not examine local control rates. Prior conventional radiation studies indicate high rates of retreatment following a single fraction of 8 Gy [12,13]. A review published in 2020 [14] listed the prescribed doses for de novo spine metastases ranging from 12 Gy to 25 Gy in a single fraction, with local control rates of 84–95%. The authors stated that treatment schedules with higher doses per fraction likely provide a higher rate of durable local control but may also carry a higher risk of toxicity. A randomized multicenter study of 13 hospitals in Canada and 5 in Australia compared SSRS (24 Gy in two fractions) to conventional radiotherapy (20 Gy in five fractions). The SSRS group showed a better pain response at 3 and 6 months. However, the median follow-up was only 6.7 months, which limits the ability to draw conclusions about long-term local control [15].

Post-SSRS Vertebral Compression Fractures

The treatment of spine metastases is primarily palliative, with most studies focusing on pain response while often overlooking treatment-related complications. Sahgal et al. [16] conducted a study addressing these concerns. They found that, while the risk of myelopathy remains low even at higher SSRS doses, the incidence of vertebral compression fractures is significantly higher. Specifically, the rates of vertebral compression fractures were exceedingly high, reaching as high as 40% for patients receiving 24 Gy in a single fraction. This risk is four times higher compared to doses lower than 20 Gy in a single fraction. Most of these fractures occurred within three months following SSRS. Although the study did not specifically address whether these fractures were symptomatic, it reported that 43% of the patients with fractures required salvage procedures, such as cement injection or instrumented stabilization. This highlights the need to balance effective pain management with SSRS with the potential for significant complications in the treatment of spinal metastases. Moreover, the risk of VCFs at adjacent vertebrae is significantly associated with increased radiation dose. A retrospective review of 239 lesions treated with single-fraction SRS revealed that the median dose for adjacent-level fractured endplates was 23.3 Gy, whereas the median dose to non-fractured endplates immediately adjacent to the SRS site was 19.1 Gy [17].

Post-Radiation Peripheral Neuropathy

Peripheral post-radiation neuropathy can be particularly debilitating, causing severe pain, motor deficits, or paresthesia, with limited treatment options. This condition is even worse in the presence of chemotherapy. High-dose single-fraction treatment has been reported to result in peripheral nervous system injury in 4.5% of patients treated in either the cervical or lumbar regions [18].

2024 ESTRO Guidelines

The recently published ESTRO guidelines [2] strongly recommend treating spinal metastatic disease with doses greater than 18 Gy when delivering a single dose. However, a notable gap in this recommendation is that the supporting data are considered to be of moderate quality despite the strong level of the recommendation. The authors also advise against using 16–18 Gy SSRS for patients with solid spinal metastases whose goals are pain control or quality of life, again providing a strong recommendation based on moderate evidence. Indeed, only 6% of the National Comprehensive Cancer Network (NCCN) guideline’s recommendations are based on level I evidence [19]. For this reason, treatment goals should be individualized depending on the patient’s aims and concerns, and should be based on an honest discussion about the risk–benefit qualities of increased radiation dose against the severity of potential complications, such as VCFs, myelopathy, and peripheral neuropathy. The current guidelines may underestimate the debilitating effects of adverse reactions associated with increased doses and do not address this issue impactfully.

Physicians should exercise caution in prioritizing local control as the sole metric of success, often at the expense of quality of life. Tumor recurrence is typically viewed as a treatment failure, while treatment-related side effects are frequently accepted as an unavoidable aspect of oncologic therapy [20,21]. This perspective can contribute to the tendency of some oncological caregivers to favor aggressive treatment strategies, potentially leading to overtreatment and increased patient morbidity [22]. In contrast to the current guidelines, treatment regimens of 16–18 Gy in a single fraction, as detailed in this paper and supported by other studies [3,23,24,25,26], have demonstrated high rates of local control coupled with low rates of complications.

### Study Limitations

This study is retrospective in nature and involves a small cohort, which limits its ability to definitively challenge the established guidelines. Our research relies on data of similar evidence quality to that which informs the guidelines, and, thus, it is not aimed at standing against them. Instead, we seek to highlight the discrepancy between the strength of the guidelines and the level of evidence supporting them. We call into question whether the guidelines adequately reflect the inherent uncertainties in the available data and suggest the need for a more nuanced approach in their construction.

## 5. Conclusions

SSRS dose escalation may increase local control efficacy but comes with a higher risk of complications. The evidence supporting the strong recommendations in the recent ESTRO guidelines is not robust enough to justify a universal application. Given the palliative nature of treatment for metastatic patients, dose determination should be individualized based on patient conditions and preferences, with a detailed discussion about the risk–benefit ratio of increased doses and the level of evidence supporting these recommendations.

## Figures and Tables

**Table 1:** Local failure vs. local control demographics (#—CHI squared value, *—Statistically significant).

		Local Failure *n*, (%)	Local Control *n*, (%)	*p* Value
Number, %		16 (6.7)	226 (93.3)	
Male gender (No., %)		7, 43%	81, 61%	0.66
Age Mean ± SD		50.9 ± 22	57.1 ± 18	0.9
Number of levels Mean, range		1.62, 1–4	1.63, 1–4	0.5
Surgery before SRS (No, %)		5, 42%	51, 51%	0.72
Previous radiation (No., %)		5, 31%	43, 32%	0.9
Involved levels	Cervical	5, 31%	29, 21.8%	0.73 #
	Thoracic	5, 31%	64, 48.1%	
	Lumbar	6, 38%	34, 25.6%	
	Sacral	0, 0%	6, 4.5%	
Pathology	Metastases	7, 43.8%	92, 69.2%	0.67 #
	Primary bone tumor	5, 31.2%	19, 14.3%	
	Nerve sheath tumor,	4, 25%	18, 13.5%	
	Meningioma			
	Misc.	0, 0%	4, 3%	
Involved region	Vertebral body	8, 53.3%	77, 58.8%	0.23 #
	Posterior elements	1, 6.7%	3, 2.3%	
	Lateral elements	3, 20%	10, 7.6%	
	Circumferential	0, 0%	13, 9.9	
	Intradural	2, 13.3%	15, 11.5%	
	Foraminal	1, 6.7%	13, 9.9	
Mean dose, range (Gy)		17.6, 16–18	17.5, 12–18	0.53
Coverage percentage (% ± SD)		92.6 ± 4.8	91.9 ± 5.4	0.8
Follow-up duration (months ± SD)		40.1 ± 30	26 ± 26	0.005 *
Local recurrence average duration, range (months)		13.9, 3–36	-	
Deceased, time post-treatment (months)		2 (12.5), 7	24 (18), 15.1	

## Data Availability

Study data is not available publicly.
